# Ultrasound super-resolution imaging for the differential diagnosis of thyroid nodules: A pilot study

**DOI:** 10.3389/fonc.2022.978164

**Published:** 2022-10-27

**Authors:** Ge Zhang, Jing Yu, Yu-Meng Lei, Jun-Rui Hu, Hai-Man Hu, Sevan Harput, Zhen-Zhong Guo, Xin-Wu Cui, Hua-Rong Ye

**Affiliations:** ^1^ Department of Medical Ultrasound, China Resources & Wisco General Hospital, Wuhan University of Science and Technology, Wuhan, China; ^2^ Hubei Province Key Laboratory of Occupational Hazard Identification and Control, Wuhan University of science and technology, Wuhan, China; ^3^ Department of Chemistry and Chemical Engineering, Queen’s University Belfast, Belfast, United Kingdom; ^4^ Department of Electrical and Electronic Engineering, Hubei University of Technology, Wuhan, China; ^5^ Department of Electrical and Electronic Engineering, London South Bank University, London, United Kingdom; ^6^ Department of Medical Ultrasound, Tongji Hospital, Tongji Medical College, Huazhong University of Science and Technology, Wuhan, China

**Keywords:** thyroid nodule, super-resolution, ultrasound imaging, microbubble, contrast-enhanced ultrasound

## Abstract

**Objective:**

Ultrasound imaging provides a fast and safe examination of thyroid nodules. Recently, the introduction of super-resolution imaging technique shows the capability of breaking the Ultrasound diffraction limit in imaging the micro-vessels. The aim of this study was to evaluate its feasibility and value for the differentiation of thyroid nodules.

**Methods:**

In this study, B-mode, contrast-enhanced ultrasound, and color Doppler flow imaging examinations were performed on thyroid nodules in 24 patients. Super-resolution imaging was performed to visualize the microvasculature with finer details. Microvascular flow rate (MFR) and micro-vessel density (MVD) within thyroid nodules were computed. The MFR and MVD were used to differentiate the benign and malignant thyroid nodules with pathological results as a gold standard.

**Results:**

Super-resolution imaging (SRI) technique can be successfully applied on human thyroid nodules to visualize the microvasculature with finer details and obtain the useful clinical information MVD and MFR to help differential diagnosis. The results suggested that the mean value of the MFR within benign thyroid nodule was 16.76 ± 6.82 mm/s whereas that within malignant thyroid was 9.86 ± 4.54 mm/s. The mean value of the MVD within benign thyroid was 0.78 while the value for malignant thyroid region was 0.59. MFR and MVD within the benign thyroid nodules were significantly higher than those within the malignant thyroid nodules respectively (*p* < 0.01).

**Conclusions:**

This study demonstrates the feasibility of ultrasound super-resolution imaging to show micro-vessels of human thyroid nodules *via* a clinical ultrasound platform. The important imaging markers, such as MVD and MFR, can be derived from SRI to provide more useful clinical information. It has the potential to be a new tool for aiding differential diagnosis of thyroid nodules.

## Introduction

Thyroid nodules are very common all over the world (1, 2). The main objective of the thyroid nodules-diagnosis is to distinguish malignant nodules from benign ones. Ultrasound (US) is a primary medical imaging tool in determining the risk stratification of thyroid nodules, which is critical for clinical management of thyroid nodules, which offers guidance for the application of fine-needle aspiration (FNA), as well as treatment decisions in patients with thyroid nodules (3, 4). Malignant US imaging patterns of thyroid nodules including solid composition, ill-defined margins, hypoechogenicity, microcalcifications, taller than wide and a lack of “halo” have been widely defined in previous studies (5).

Thyroid Imaging Reporting and Data System (TI-RADS), as the most used ultrasound-based malignancy risk stratification systems of thyroid nodules, is not enough to exclude or confirm thyroid malignancy sensitively and specifically (6, 7). TI-RADS classification is largely affected by the inter-observer variability, which results in suboptimal sensitivity and specificity (8). Furthermore, atypical benign and malignant nodules, especially TI-RADS 3 and 4 nodules, have a certain overlap in patterns during the routine US examination. These overlaps can easily lead to misdiagnosis of diseases and thus challenges for clinicians. Therefore, it is particularly important to explore a new way to accurately diagnose benign and malignant nodules.

Vascular distribution and flow characteristics within the nodule are broadly believed to play an important role in determining tumor characteristics (9). A number of scholars believe that angiogenesis has a great importance for distinguishing benign and malignant thyroid nodules. Micro-vessel density (MVD) has been regarded as the gold standard for the evaluation of tumor angiogenesis (10). Furthermore, some studies have indicated that counting micro-vessels may reflect local extrathyroidal and vascular invasion (11). Jiang performed immunohistochemical staining for CD31 and CD34 to obtain MVD on 122 thyroid nodules with different pathological types. The results showed the MVD in benign thyroid nodules was significantly higher than that in malignant thyroid nodules (12). Considering the invasiveness of acquiring MVD in this way, a new method to obtain micro-vessel density in thyroid nodules non-invasively still requires further exploration.

Color Doppler flow imaging (CDFI) is a widely used US blood flow imaging since the motion of red blood cells acting as scatterers. However, only relatively fast flow (larger than 1 cm/s) in the large vessels can be detected (13). Thus, CDFI is not an ideal imaging approach to observe the microvasculature and also the relatively slow flows in these micro-vessels. In the last several decades, the application of contrast-enhanced ultrasound (CEUS) on thyroid-related diseases has greatly improved. CEUS can significantly enhance the US contrast echo of the bloodstream, nevertheless, the imaging resolution of CEUS is still limited by the diffraction limit of US applied (14). Therefore, CEUS is still insufficient to observe the microvasculature at the capillary level and also the microvascular flow rate (MFR) within the micro-vessels. Besides, no guidelines recommend CDFI and CEUS as a routine method for US risk stratification of thyroid nodules (15–17).

In the last few years, inspired by optical super-resolution imaging (SRI), US SRI technique can bypass the compromise between penetration and resolution as the conventional US imaging is limited in image resolution by the diffraction to the scale of wavelength. After the injection of a low concentration of microbubble contrast agents, spatially isolated microbubbles can be localized, and their displacement can be tracked within a subwavelength resolution (18, 19). Therefore, US SRI and super-resolved velocity map (SRVM) can be generated at such a submicron scale, providing possibility to bridge the gap between multiple imaging techniques and histopathology (20, 21). After the generation of US SRI and SRVM, a number of useful clinical parameters could be derived from them, such as MVD and MFR. These clinical parameters could effectively help clinicians for clinical decision-making and also the disease managements. US SRI was firstly demonstrated *in vitro* using a single micro-channel in 2011 to show that US SRI could remarkably break the inherent diffraction limit (22). US SRI technique was then implemented in the mouse ear in 2015. This study firstly introduced the concept of SRVM and showed that the magnitude and direction of flow velocity within the microvasculature can be derived (23). Moreover, US SRI was demonstrated in the rat brain (18) and a cancer model (24) respectively to show its potential to be a useful tool for a more comprehensive understanding and diagnostics of various disease progressions that alter the microvascular blood flow.

To our best knowledge, there have been no studies on the performance of SRI in discriminating between benign and malignant thyroid nodules. This study intended to explore whether SRI technique could image micro-vessels in thyroid nodules. Based on this, the CEUS images obtained by a low concentration of microbubble injection were used to perform US SRI processing. MFR and MVD within the thyroid area were further calculated to probe a new technical method for distinguishing benign and malignant thyroid nodules.

## Materials and methods

### Clinical data acquisition

From November 2021 to December 2021, 24 patients with thyroid nodules were enrolled (comprising 24 thyroid nodules that were classified as TI-RADS category 3 or 4). Patients aged ≥ 18 years with solid thyroid nodules that were > 5 mm in maximum diameter can be included in the study. Potential patients with any of the following conditions: large calcifications of the nodule with posterior acoustic shadow; previous medication, radiation, or surgery of the thyroid; hyperthyroidism, thyroiditis; or currently pregnant were excluded. All 24 thyroid nodules in 24 patients were diagnosed based on histopathological results from surgical excision or FNA. This study was approved by the Institutional Review Board of China Resources & Wisco General Hospital (approval number: HRWGZYY20210021). Written informed consent was obtained from each patient who underwent CEUS examinations.

All the ultrasound examinations were conducted by one senior radiologist who has received standardized training of thyroid nodules before collecting materials in this study. All patients underwent B-mode, CDFI and CEUS imaging respectively with an US platform (Resona 9s, Mindray Bio-Medical Electronics Co. Ltd., Shenzhen, China) and an L14-5WU linear array transducer (9.0 MHz center frequency, bandwidth 4 MHz - 14 MHz). During the scanning, the patients were asked to be supine and to expose the neck fully. The thyroid nodules were scanned in transverse and longitudinal sections. The patients were asked to avoid swallowing and hold their breath briefly during the scan to avoid the motion. According to the TI-RADS classification, the location, size, composition, echogenicity, margins, calcifications, and shape of thyroid nodules were observed and recorded, and then the nodules were assigned into different TI-RADS categories according to their total scores. Meanwhile, CDFI was also conducted to observe the blood flow and vascular morphology in and around the nodules. All the image datasets were acquired respectively at the same plane with the most abundant blood supply in each nodule.

For the SRI data acquisition, a low concentration of SonoVue (Bracco, Milan, Italy) microbubble solution was injected intravenously as a bolus of 0.1 mL as this technique requires to localize spatially isolated individual microbubble signals. Real-time dual-mode images (B-mode and CEUS) were utilized to guide the image plane and observe the microbubble signals after the injection. For each dataset, more than 1,500 images were acquired at a frame rate of 80 Hz. A mechanical index (MI) of 0.08 was used to avoid the microbubble destruction during the CEUS examinations. The other imaging parameters were set on the US system as follows: gain 10 dB, dynamic range 100. A stack of several thousands of US images was obtained for thyroid nodules for further US SRI processing. For each dataset, the pathological results received from biopsy and quantifications achieved from US SRI were compared.

**Figure 1:**
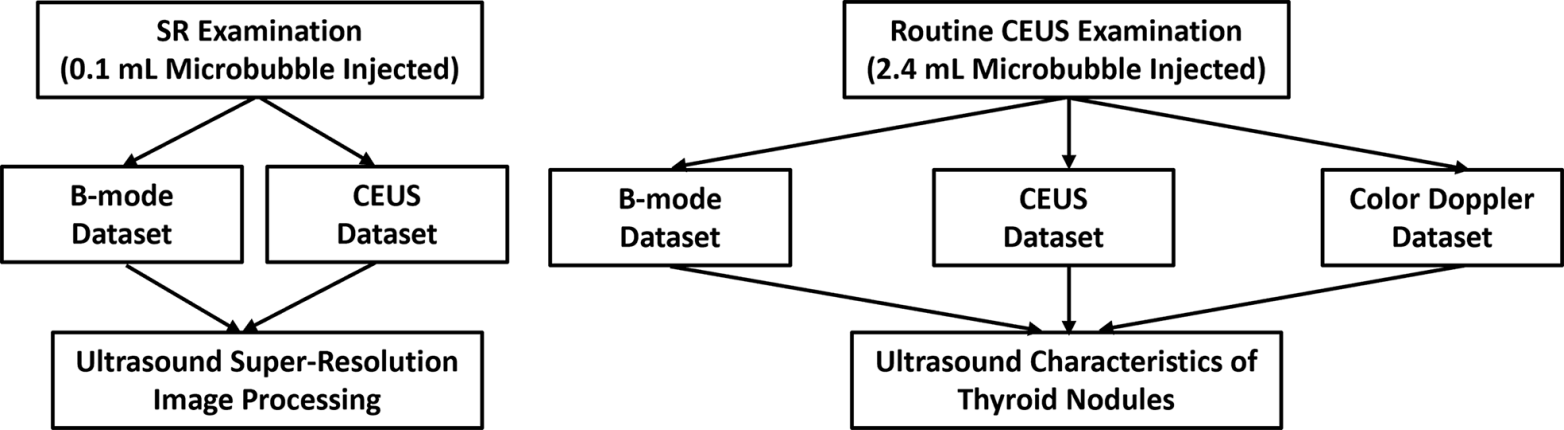
Data acquisition pipeline. A low concentration of microbubbles (0.1 mL) was injected to acquire B-mode and CEUS datasets for further US SRI processing. Then a routine concentration of microbubbles (2.4 mL) was injected to acquire B-mode, CEUS and CDFI datasets for US characteristics of thyroid nodules.

For the routine CEUS examination, 2.4 mL of SonoVue was rapidly injected intravenously followed by a 5 mL of physiological saline solution to flush and promote the perfusion of the contrast agent in the blood vessels. The other imaging parameters remained the same as the previous SR examination. The data acquisition pipeline is shown in **Figure 1**.

### Ultrasound imaging processing

The image data was obtained *via* the clinical US platform. The super-localization processing was performed offline using MATLAB (version R2021b; MathWorks Inc., Natick, MA, USA). A previous established two-stage motion correction (25–27) was applied to correct the tissue motion during the scanning.

For each dataset, a singular value decomposition (SVD) processing technique was used to filter out the tissue signal and retain the microbubble signals. Super-Localization processing was performed on each frame after setting an image pixel value threshold to reject the noise and detect potential microbubble signals. Each observed point spread function (PSF) was compared with a calibration PSF according to their area (A), intensity (I), and shape/eccentricity (E). These parameters were used to discard potential non-microbubble signals and noises. All the observed PSFs with the corresponding three attributes were summarized into three matrices. All the values were normalized in each matrix. The location of each spatially isolated microbubbles was calculated by the “centroid” method. The centroid of each localized microbubble was computed by its intensity-weighted center of mass. All the localizations from all the images were assembled into the final SRI (28).

The MFR was calculated based on the region of interest (ROI) which was manually drawn on MATLAB referring to the contours of benign and malignant thyroid nodules on both the B-mode image and the corresponding SRVM. To compute the MFR, the tracking method computes the best correlated bubble signals within a search window between neighboring images. Briefly, each microbubble detected in the image H and each of the microbubbles in the image H+1 was recorded within a search window. Since the frame rated of 80 Hz was used, 800 micrometers was set as the maximum search window so that flow rate up to 15 mm/s can be tracked. For each signal in the frame H, a paired signal in the image H+1 was identified if they have the maximum normalized cross-correlation value above a determined threshold of 0.8. The distribution of microvascular flow rate was visualized by the histogram of microbubble velocities.

**Figure 2:**
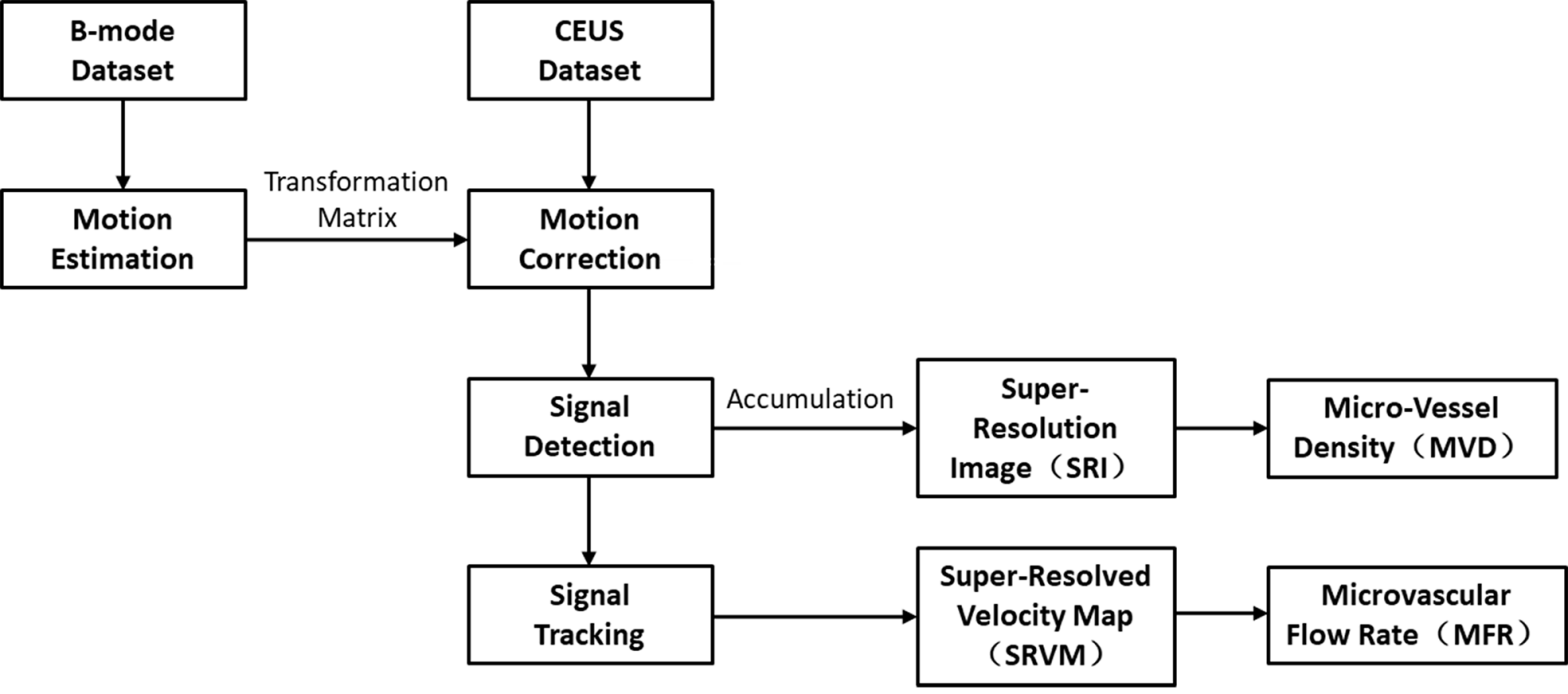
Post-processing flow chart to compute US SRI and the corresponding parameters. Motion estimation is performed on the original B-mode dataset, the generated transformation matrix is applied on the corresponding CEUS dataset to compensate for the movements. Then the bubble signals are detected and tracked over frames to compute the final SR images.

The MVD was defined as tracked microbubble area divided by the ROI area. The ROI was also manually drawn on MATLAB referring to the contours of benign and malignant thyroid nodules on both the B-mode image and the corresponding SRI. The post-processing flow chart used in this study is shown in **Figure 2**.

### Statistical analysis

Results were expressed as the mean ± standard deviation. Statistical significance was analyzed using the two-sample t-test. All the continuous variables conformed to a normal distribution. A chi-square test was performed for independence to observe if two variables are related. A p value smaller than 0.05 was regarded to suggest statistical significance; a p-value smaller than 0.01, strong significance; and a p value smaller than 0.001, high significance.

## Results

### Ultrasound images of thyroid nodules

As shown in **Table 1**, clinical information, and grayscale US characteristics of 24 thyroid nodules who underwent thyroid imaging from the China Resources & WISCO General Hospital medical records database (12 cases in each group of benign and malignant groups) were obtained. 24 TI-RADS category 3 and 4 thyroid nodules were identified as 12 malignant thyroid nodules and 12 benign nodules *via* the pathological results; all malignant nodules were papillary carcinomas (n = 12). Benign nodules included: thyroid adenoma (n = 6), nodular goiter (n = 5), and 1 other (subacute thyroiditis, n = 1). The gender, age, nodule size and composition were not found to differ significantly between malignant and benign nodules (*p* > 0.05). Among the sonographic features, hypoechogenicity or marked hypoechogenicity and microcalcification and aspect ratio had statistic differences between malignant and benign nodules (*p* < 0.05).

**Table 1:** Patient clinical information and grayscale US characteristics of thyroid nodules for 24 patients.

Characteristic	Benign	Malignant
Number of nodules	12	12
Age#	50.9 (31-82)	44.7 (29-53)
Gender		
Female n (%)	8 (66.7)	9 (75.0)
Male	4 (33.3)	3 (25.0)
Size		
≤1cm	5 (41.7)	6 (50.0)
1-2cm	5 (41.7)	5 (41.7)
>2cm	2 (16.7)	1 (8.3)
Composition		
Cystic	0 (0.0)	0 (0.0)
Sponge-like	0 (0.0)	0 (0.0)
Mixed	0 (0.0)	0 (0.0)
Solid	12 (100)	12 (100)
Echogenicity		
Anechogenicity	0 (0.0)	0 (0.0)
Iso- or hyperechogenicity	4 (33.3)	0 (0.0)
Hypoechogenicity	8 (66.7)	4 (33.3)
Marked hypoechogenicity	0 (0.0)	8 (66.7)
Shape		
Wider than tall	12 (100)	7 (58.3)
Taller than wide	0 (0.0)	5 (41.7)
Microcalcifcation		
Microcalcifcation	0 (0.0)	9 (75.0)
No microcalcifcation	12 (100)	3 (25.0)
Margin		
Well defined	9 (75.0)	0 (0.0)
Poorly defined	1 (8.3)	7 (58.3)
Irregularity or lobuling	1 (8.3)	2 (16.7)
Extracapsular spread	1 (8.3)	3 (25.0)
TI-RADS classification		
TI-RADS 3	6 (50.0)	1 (8.3)
TI-RADS 4	6 (50.0)	11 (91.7)

unless otherwise specified, data in parentheses are percentages. ^#^Numbers in parentheses are the range.

**Figure 3:**
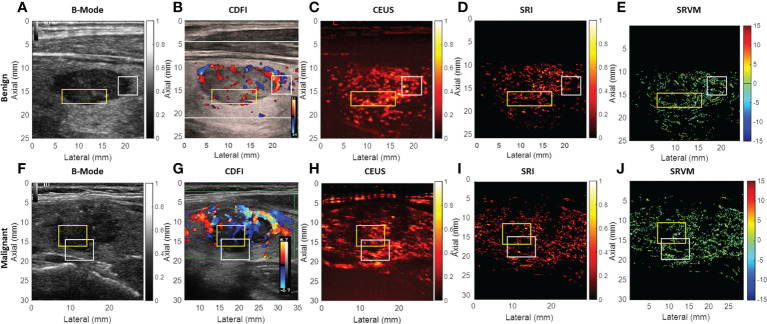
US images of the representative benign and malignant thyroid nodules. **(A)** B-mode image of the benign thyroid nodule. **(B)** CDFI images show the flow within the benign thyroid nodules. **(C)** CEUS image shows the accumulation of microbubbles along the frames within the benign thyroid nodule. **(D)** SRI shows the microvasculature within the benign thyroid nodule. **(E)** SRVM shows the flow velocity within the benign thyroid nodule. **(F)** B-mode image of the malignant thyroid nodule. **(G)** CDFI images show the flow within the malignant thyroid nodules. **(H)** CEUS image shows the accumulation of microbubbles along the frames within the malignant thyroid nodule. **(I)** SRI shows the microvasculature within the malignant thyroid nodule. **(J)** SRVM shows the flow velocity within the malignant thyroid nodule.

**Figure 4:**
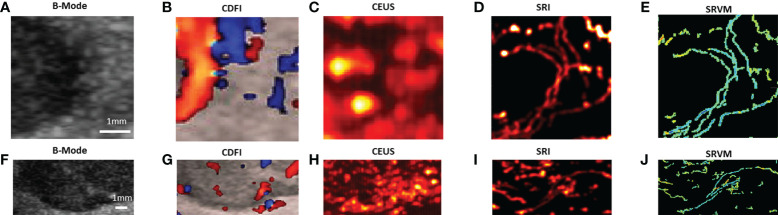
Two zoomed-in sections showing the detailed comparisons of benign thyroid nodule between **(A, F)** B-mode, **(B, G)** CDFI, **(C, H)** CEUS, **(D, I)** SRI and **(E, J)** SRVM as the white and yellow boxes indicated in **Figures 3A–E**.

**Figure 5:**
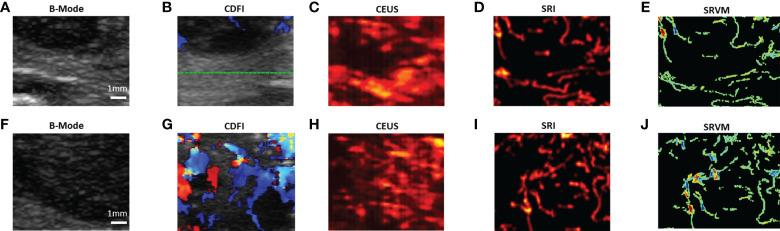
Two zoomed-in sections showing the detailed comparisons of malignant thyroid nodule between **(A, F)** B-mode, **(B, G)** CDFI, **(C, H)** CEUS, **(D, I)** SRI and **(E, J)** SRVM as the white and yellow boxes indicated in **Figures 3F–J**.

Various imaging techniques were performed to depict the microvasculature of benign and malignant thyroid nodules on human. SRI was also performed to visualize the microvasculature. Each moving isolated microbubble in the representative benign and malignant thyroid nodules was tracked locally to generate SRI and super-resolved velocity map (**Videos 1**, **2**). **Figure 3** above showed the B-mode and the corresponding CDFI, CEUS and SRI and SRVM images of the representative benign (A-E) and malignant (F-J) thyroid nodule respectively. **Figure 4** showed two zoomed-in sections of the benign thyroid nodule in **Figures 3A–E** as the white and yellow boxes indicated in **Figures 3A–E** respectively. **Figure 5** showed two zoomed-in sections of the malignant thyroid nodule in **Figures 3F–J** as the white and yellow boxes indicated in **Figures 3F-J** respectively.

For CDFI, various flow patterns can be observed in both benign and malignant thyroid nodules, which include spotty, short lines, branching, and tortuous. Moreover, only large vessels with relatively fast blood flows can be observed as CDFI technique was not sensitive enough to visualize the microvascular flow. However, SRVM can visualize the microvascular flow with finer details and reveal the MFR within the microvasculature clearly whereas CDFI cannot as demonstrated in **Figures 4B, E, G, J** and **5B, E, G, J**. Similar to CDFI, in the SRVM, the red color represented the relatively high flow rate and blue color represented the relatively low flow rate. It should be noted that a number of microvascular flow patterns shown in SRVM cannot be observed in CDFI.

For CEUS, after the injection of microbubble solution, the CEUS images showed an iso-enhancement pattern in the benign thyroid nodule and a heterogeneous hypo-enhancement pattern in malignant thyroid nodule. Microvasculature cannot be clearly seen on CEUS since the image resolution is inherently limited by the US transmission frequency. After super-localization processing, SRI can reveal the microvasculature within the thyroid nodule with much greater detail. Two adjacent micro-vessels cannot be observed on CEUS whereas they can be clearly seen in SRI as demonstrated in **Figures 4C, D** and **5C, D**. Additionally, the tortuosity of individual micro-vessels was clearly reflected in SRI as SRI offered a better image resolution which could distinguish adjacent micro-vessels. However, it is challenging to observe tortuosity of individual micro-vessels in CEUS as the contrast signal of two adjacent micro-vessels may overlap together into a large vessel as shown in (**Figures 4C, D, H, I** and **5C, D, H, I**).

### Quantification of ultrasound super-resolution imaging

**Figure 6:**
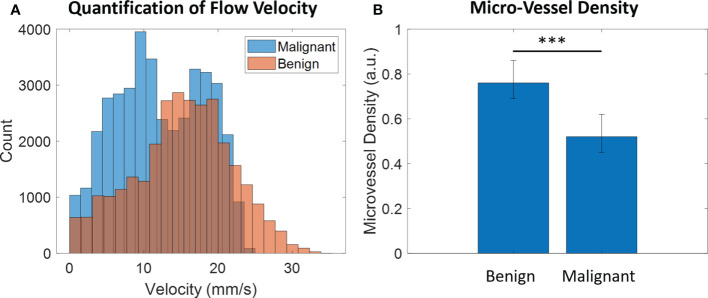
Quantification of **(A)** MFR and **(B)** MVD in both benign and malignant thyroid nodules. *** represents that there is a highly significant difference between two groups (p-value < 0.001).

The MFR and MVD in both benign and malignant thyroid nodules were quantified respectively as shown in **Figure 6** and **Table 2**. The mean value of the MFR in benign thyroid nodule region was 16.76 ± 6.82 mm/s whereas that in malignant thyroid region was 9.86 ± 4.54 mm/s. The mean value of the MVD in benign thyroid region was 0.78 while the value for malignant thyroid region was 0.59. A chi-square test was performed for independence to observe if two variables are related. A p value of < 0.001 suggests that two variables are related. The results showed that MFR and MVD within the benign thyroid nodules were 41.2% and 24.4% significantly higher than those within the malignant thyroid nodules respectively.

**Table 2:** Chi-square test for MVD and MFR.

Parameters	Mean (Benign)	Std. (Benign)	Mean (Malignant)	Std. (Malignant)	Chi-Square	*p*
**MVD**	0.78	0.12	0.59	0.10	56.87	<0.001
**MFR**	16.76	6.82	9.86	4.54	98.55	<0.001

## Discussion

In this study, we utilized a novel US SRI technique to visualize the microvasculature and the corresponding microvascular flow rate within thyroid nodules. Compared with conventional CDFI and CEUS modalities, SRI could reveal more blood flow information within thyroid nodules; our results demonstrate the feasibility of using SRI to visualize microvasculature in human thyroid nodules. In addition, US SRI is capable of generating maps of microvascular morphology and blood flow velocity with a capillary-level image resolution. These features allow for a detailed analysis of local hemodynamics within the thyroid region, showing that the MFR and MVD within benign thyroid nodules are significantly higher than those within malignant thyroid nodules. This work may provide new important imaging markers for clinicians to differentiate benign and malignant thyroid nodules based on the microhemodynamics of thyroid nodules. For the first time as far as we are aware, US SRI technique was applied to help differentiate the benign and malignant thyroid nodules in patients with a clinically available US system and scanner. Image features, such as MFR and MVD, computed from the US SRI were evaluated to differentiate the benign and malignant thyroid nodules.

Previous studies examining vascularity within thyroid nodules by CDFI to differentiate benign from malignant thyroid nodules have shown controversial results. By analyzing 698 thyroid nodules of multiple studies, the results showed that 55.56% of the studies showed increased vascularity in malignant thyroid nodules, while the remaining studies showed no difference or even a decrease (29). The possible reasons for this discrepancy may be the differences in nodule size and pathological type. These different manifestations of vascularity in the benign and malignant thyroid nodules limit the application of CDFI in the differentiation of benign and malignant nodules. Our results demonstrated no significant difference was showed in blood flow patterns between benign and malignant thyroid nodules as showed in **Figure 3**. This diagnostic variability may be since CDFI cannot overcome the diffraction limit of ultrasound to truly reveal intra-nodular microvascular information (14).

CEUS makes up for deficiencies of conventional B-mode ultrasound and CDFI for imaging various diseases (30). The main adding value of CEUS is the visualization of sequence and intensity of vascular perfusion and hemodynamics. However, to date, no specific contrast enhancement pattern can be used to diagnose benign and malignant thyroid nodules alone (31). Widely accepted CEUS enhancement patterns for diagnosing malignant thyroid nodules include the following criterion: hypo-enhancement, heterogeneous and slow wash-in and wash-out curve lower than in normal thyroid tissue. The pathological basis may be related to the malignant nodules’ complex neovascularization. Once the tumor grows beyond the newly formed vasculature, necrosis and embolism may occur in the tumor, resulting in an uneven enhancement or low enhancement pattern (5, 12, 32). Combining CEUS and quantitative analysis, our results also showed that malignant thyroid nodules tend to have low enhancement or uneven enhancement but without statistical difference. This may be due to the small sample size. Despite the high sensitivity and specificity of CEUS in diagnosing nodules, there is still a 12.5% missed diagnosis rate and a 13.67% misdiagnosis rate (5). Thus, a new method to obtain vascular distribution and flow characteristics in thyroid nodules non-invasively still requires further exploration.

US SRI has shown the capability to noninvasively visualize and quantify microvascular structures and blood flow dynamics with resolution below the wave diffraction limit (33–35). A large number of previous studies have demonstrated the capability of applying US SRI technique *in-vitro* and in various animals (23, 28, 34, 36). Recently, several studies have shown that, US SRI technique can be successfully applied on human brain and kidney to further help clinicians for medical diagnosis (37–39). In 2018, first-in-human applications of the US SRI techniques using clinical scanners were demonstrated in human breast cancer (40). Opacic et al. showed that US SRI could provide a number of clinical parameters such as velocities and directions of movement in individual vessels which can differentiate tumors with different vascular phenotypes, and it may provide opportunities for functional characterization of tumors and the assessment of therapy response. To assess any early changes in breast cancer following vascular disrupting agent (VDA) treatment, a study using 3-dimensional (3-D) super-resolution ultrasound (SR-US) imaging to observe MVD in breast lesions showed that the MVD of each tumor volume was significantly reduced at 24 hours after VDA treatment (41), indicating the feasibility of super-resolution technique for disease diagnosis *via* calculating micro-vessel density and microvascular flow rate in masses. However, there is no previous study reported the application of US SRI on human thyroid nodule to differentiate the benign and malignancy. In this study, our results confirmed that the super-resolution technique can not only clearly display the micro-vessels in thyroid nodules, but also quantify the MFR and MVD in the nodules. In addition, the MFR and MVD within benign thyroid nodules were significantly higher than those within malignant thyroid nodules, which showed the consistency with the previous study. The possible explanations could be that, in malignant thyroid region, the micro-vessels are more irregular and tortuous. Previous study has also reported that, some malignant thyroid nodules are completely avascular (42).

There are a few challenges existing in the general SRI technique. A previous study has reported a successful two-stage motion correction technique on the super-resolution imaging of human lower limb (28). This study has shown that two-stage motion correction could significantly correct the in-plane motion for the super-resolution imaging. For the out-of-plane motion, it is still a challenge for the 2D US imaging. Therefore, a 3D super-resolution imaging technique equipped with a 2D array probe is expected to overcome this problem. It should be noted that, the CDFI shown in **Figure 3** were not acquired at the same time as the CEUS image acquisition. Therefore, it may contribute to the reason that the CDFI images were not well corresponded to the final SR image. Another challenge exists in this study is that the acquisition of CEUS images for SR image processing requires the patients and the image transducer to remain stationary during the image acquisition. This is another general challenge for all the SR image processing (43). This is because it is difficult to ask patients to hold their breath for more than 10 s to acquire the image data. Moreover, heart beating is another inevitable factor that contributes to the tissue motion. All these factors would affect the SRI reconstruction of microvasculature.

There are some other limitations existing in this study. First, the sample size is relatively small in this study. Nevertheless, multiple studies have demonstrated the feasibility and reliability of super-resolution techniques to provide a number of clinical parameters of micro-vessels at the micron scale. A larger sample size is desired to draw a more solid conclusion to differentiate the benign and malignant thyroid nodules using US SRI. SR image generation is quite time-consuming and requires offline processing. The graphics processing unit (GPU) parallel processing is also desired to be integrated into the SR processing in the future to further accelerate the SR image generation. Second, the MVD in benign and malignant nodules could not be obtained by immunohistochemical staining in this study, however the difference in MVD obtained by SRI in benign and malignant thyroid nodules showed consistency with previous study (12), manifesting that the MVD within the benign thyroid nodules were significantly higher than those within the malignant thyroid nodules.

In conclusion, US SRI has been successfully demonstrated on human thyroid nodule to show finer microvascular details at a submicron scale image resolution and obtain important imaging markers, such as MFR and MVD within the thyroid nodule region which cannot be obtained from conventional B-mode, CEUS and CDFI techniques. Conventional multimodal US imaging techniques together with SRI technique could effectively improve the diagnostic accuracy in differentiating benign and malignant thyroid nodules as more useful clinical information (such as MVD and MFR parameters) can be provided for radiologists to differentiate benign and malignant thyroid nodules. The gap between conventional imaging techniques and histopathology can be bridged *via* US SRI to provide a new approach for clinicians to manage thyroid nodules and avoid unnecessary punctures.

## Data availability statement

The raw data supporting the conclusions of this article will be made available by the authors, without undue reservation.

## Ethics statement

The studies involving human participants were reviewed and approved by The Institutional Review Board of China Resources & Wisco General Hospital. The patients/participants provided their written informed consent to participate in this study.

## Author contributions

GZ, JY, Y-ML, J-RH, M-HY, HC, NL, and H-MH contributed to the collection of relevant literature. Sevan Harput contributed the technical support of SRI technique. GZ and JY contributed to the literature analysis and manuscript preparation. GZ and JY sorted out the literature and wrote the manuscript. Z-ZG, X-WC and H-RY were responsible for design of this article and provided data acquisition, analysis, and interpretation. All authors contributed to the article and approved the submitted version.

## Funding

This work was supported by the Key Research and Development Project of Hubei Province (No.2020BCB022), the National Natural Science Foundation of China (No. 82071953), the joint fund project of the Hubei Provincial Health and Family Planning Commission (No.WJ2019H197) and Hubei Province Key Laboratory of Occupational Hazard Identification and Control, Wuhan University of science and technology (No: 0HIC2019G08).

## Acknowledgments

The authors would like to thank the help from Prof. Mengxing Tang at Imperial College London in the completion of this article and the reviewers for reviewing this article. Further, we also would like to thank the help from M-H Y, HC and NL for the collection of relevant literature.

## Conflict of interest

The authors declare that the research was conducted in the absence of any commercial or financial relationships that could be construed as a potential conflict of interest.

## Publisher’s note

All claims expressed in this article are solely those of the authors and do not necessarily represent those of their affiliated organizations, or those of the publisher, the editors and the reviewers. Any product that may be evaluated in this article, or claim that may be made by its manufacturer, is not guaranteed or endorsed by the publisher.
